# Subfoveal choroidal thickness and retinal nerve fiber layer alterations in chronic heart failure patients

**DOI:** 10.5935/0004-2749.20210077

**Published:** 2021

**Authors:** Hatice Selen Kanar, Aysegul Penbe, Batur Gonenc Kanar

**Affiliations:** 1 Department of Ophthalmology, Kartal Dr. Lutfi Kirdar Training and Research Hospital, Health Science University, Istanbul, Turkey; 2 Department of Cardiology, Faculty of Medicine, Marmara University, Istanbul, Turkey

**Keywords:** Heart failure/complications, Choroid/pathology, Tomography, optical coherence, Nerve fibers, Retina, Insuficiência cardíaca/complicações, Coroide/patologia, Tomografia de coerência óptica, Fibras nervosas, Retina

## Abstract

**Purpose:**

To comparatively evaluate the subfoveal choroidal thickness and the peripapillary retinal nerve fiber layer thickness in patients with chronic heart failure relative to control subjects.

**Methods:**

A total of 72 chronic heart failure patients and 40 healthy control subjects were enrolled in this study. The patients were categorized into 2 groups: group 1: patients with 30-50% left ventricle ejection fraction and group 2: patients with the corresponding fraction value of <30%. The subfoveal choroidal thickness and the peripapillary retinal nerve fiber layer thickness were measured by spectral domain-optical coherence tomography.

**Results:**

The mean subfoveal choroidal thickness was 250.24 ± 68.34 µm in group 1 and 216.72 ± 71.24 µm in group 2, while it was 273.64 ± 77.68 µm in the control group. The differences among the 3 groups were statistically significant. The average peripapillary retinal nerve fiber layer thicknesses were 100.34 ± 8.24, 95.44 ± 6.67, and 102.34 ± 8.24 µm, respectively. No significant differences were noted in the peripapillary retinal nerve fiber layer thicknesses between group 1 and control group, but it was significantly lower in group 2.

**Conclusion:**

Our study thus revealed that the subfoveal choroidal thickness was lower in patients belonging to both the chronic heart failure groups in comparison to those in the control group. However, the alteration in the peripapillary retinal nerve fiber layer thickness was noted in only patients with <30% left ventricle ejection fraction. In the clinical practice, reductions in these values are correlated with decreased left ventricle ejection fraction, which may be important for the follow-up of chorioretinal diseases and the evaluation of glaucoma risks in patients with chronic heart failures.

## Abstract (pt)

**Objetivo:**

O objetivo do nosso estudo foi avaliar a espessura coroidal subfoveal e a camada peripapilar de fibras nervosas da retina em pacientes com insuficiência cardíaca crônica, em comparação com um grupo de controle.

**Métodos:**

Setenta e dois pacientes com insuficiência cardíaca crônica e 40 controles saudáveis foram inscritos. Os pacientes com insuficiência cardíaca crônica foram divididos em dois grupos, de acordo com a fração de ejeção do ventrículo esquerdo. Pacientes com fração de ejeção do ventrículo esquerdo de 30-50% foram incluídos no grupo 1, enquanto valores de fração de ejeção do ventrículo esquerdo inferiores a 30% foram incluídos no grupo 2. A espessura coroidal subfoveal e a espessura da camada peripapilar de fibras nervosas da retina foram medidas por tomografia de coerência óptica de domínio espectral.

**Resultados:**

A espessura média da coroide subfoveal foi de 250,24 ± 68,34 µm no grupo 1, 216,72 ± 71,24 µm no grupo 2 e 273,64 ± 77,68 µm no grupo controle. As diferenças entre os três grupos foram estatisticamente significativas. A espessura média da camada peripapilar de fibras nervosas da retina foi de 100,34 ± 8,24 µm, 95,44 ± 6,67 µm e 102,34 ± 8,24 µm, respectivamente. Não houve diferença significativa na espessura da camada peripapilar de fibras nervosas da retina entre o grupo 1 e o grupo controle, mas os valores foram significativamente menores no grupo 2.

**Conclusão:**

Nosso estudo mostrou que a espessura coroidal subfoveal foi menor em ambos os grupos de insuficiência cardíaca crônica, em comparação com controles saudáveis. Porém, a camada peripapilar de fibras nervosas da retina mostrou-se alterada apenas em pacientes com menos de 30% da fração de ejeção do ventrículo esquerdo. Na prática clínica, reduções nesses valores, correlacionadas com a diminuição da fração de ejeção do ventrículo esquerdo, podem ser importantes para o acompanhamento de doenças coriorretinianas e a avaliação dos riscos de glaucoma em pacientes com insuficiência cardíaca crônica.

## INTRODUCTION

Heart failure is a pathophysiological state in which abnormality in the cardiac function can result in failure of the heart to pump the blood under normal cardiac pressures at a rate that meets the requirements of the metabolizing tissues^(1)^. The prevalence of chronic heart failure (CHF) is gradually increasing and has been related to high morbidity, mortality, and health care expenditure. In the recent decades, significant advancements have occurred in the medical and device treatment developments for heart diseases; however, the long-term prognosis of CHF generally remains poor^(2)^.

The pathomechanism of CHF is not fully understood. Some past evidence indicates that CHF is closely associated with endothelial dysfunction and involve an analogous pathophysiological process, including smoking, obesity, diabetes, and hypertension^(3,4)^. The reduction in the cardiac output in CHF is associated with the compensatory mechanisms of peripheral vasoconstriction toward sustaining sufficient blood pressure^(5)^. Several studies on eyes have reported a possible reduction in the cerebral blood circulation or in the ophthalmic arterial blood circulation; these reductions can be attributed to CHF-associated hypoperfusion^(6-8)^. The choroidal vascular structures are primarily responsible for the blood supply to the outer retinal layers, which are important in the visual pathway^(9)^. For example, the choriocapillaris, a highly fenestrated sinusoidal vascular plexus, is the site of the greatest blood flow through the body, which comprises up to 85% of the eye blood volume, and it nourishes the outer portion of the retina^(10)^. Consequently, any changes in the hemodynamic parameters associated with CHF may affect the ocular fluid dynamics as well as the composition and the blood vessels, retinal tissues, and choroid. Vasoconstriction of the retrobulbar vessels may cause instability of the blood flow in the optic nerve head, while ischemia, followed by reperfusion, is a known cause of oxidative stress and cell death due to apoptosis. The peripapillary retinal nerve layer (pRNFL) loss may therefore be considered as a secondary consequence of hypoperfusion in CHF.

The evaluation of choroidal tissues has become easier with the advent of spectral domain optical-coherence tomography (SD-OCT) technology. Several systemic diseases are related to the alterations in the subfoveal choroidal thickness (SFCT) and the pRNFL thickness (pRNFLT)^(11-13)^. This study aimed to compare the SFCT and RNFL values among patients with CHF and healthy controls.

## METHODS

This cross-sectional study was conducted at the Ophthalmology Department of the Kartal Dr. Lutfi Kirdar Training and Research Hospital and at the Cardiology Department of the Marmara University Faculty of Medicine. The study protocol followed the tenets of the Declaration of Helsinki and was approved by the local ethics committee.

Patients with CHF were diagnosed by a cardiologist with reference to the diagnostic criteria of the European Society Cardiology, Congestive Heart Failure and Treatment Guideline. The patients showing heart failure symptoms and with left ventricular ejection fraction (LVEF) <50% (as assessed by echocardiography) were accepted as CHF patients. These patients were categorized into 2 groups based on their LVEF values. The group 1 patients had 30-50% LVEF, while group 2 patients had <30% LVEF values. All CHF patients were compared with their corresponding ageand sex-matched healthy controls.

### Ophthalmic examinations

All subjects underwent a detailed ophthalmic examination that included testing for best corrected visual acuity using the Snellen chart, intraocular pressure measurement (IOP) with the Goldmann applanation tonometry, slit lamp biomicroscopy, and dilated fundus examination. The axial length (AL) was measured with the IOL Master 500 (Carl Zeiss Meditec Inc., Jena, Germany). The standard automated perimetry used the 30-2 SITA Program (Humphrey Visual Field Analyzer; Carl Zeiss Meditec, Inc., Dublin, CA, USA). The parameters of total deviation (TD), mean deviation (MD), and pattern standard deviation (PSD) were also evaluated. The mean ocular perfusion pressure (OPP) was calculated by using the following formula: mean OPP=2/3 x mean arterial blood pressure (MABP) - IOP^(14)^. The SCFT and the pRNFLT were also determined by using SD-OCT (Nikon RS-3000, Japan). All SD-OCT measurements were performed by the same technician. All SFCT data, which were determined as the axial distance from the RPE to the outer choroid/sclera interface, were assessed by the same ophthalmologist by enhanced-depth image (EDI) scanning. The pRNFLT was determined by SD-OCT using a 3.46-mm-diameter scan circle centered on the optic disc. The pRNFLT values of 4 quadrants (N - nasal, T - temporal, S - superior, and I - inferior), 6 sectors (N - nasal, NS - nasal-superior, T - temporal, TS - temporal-superior, and NI - nasal-inferior), and the global mean values (360 degrees) were obtained. The average values for all quadrants were applied to statistical analyses.

Patients with high myopia (>6D), age macular degeneration, or advanced cataracts or those with a history of retinal vascular disease, retinal dystrophy, retinal surgery, or laser photocoagulation were excluded from the study. In addition, patients with diabetes were excluded because of the potential effects on the choroidal circulation and SFCT in them.

### Cardiological examinations

The systolic blood pressure (SBP) and diastolic blood pressure (DBP) of the subjects were determined by using a sphygmomanometer with subjects in a sitting position. The readings for SBP and DBP were obtained after the subjects were seated for 10 min. The MABP was calculated according to the following formula: MABP=2/3 x DBP + 1/3 x SBP.

Echocardiographic analysis was performed by using the M mod, B mod, and two-dimensional (2D) Doppler apparatus. LVEF was quantitatively estimated by the Simpson method. The 2D echocardiographic evaluations of the cardiac chamber quantifications and the LV’s systolic function were measured by an ultrasound system (Epic; Philips Healthcare Medical Systems, Andover, Massachusetts, USA) in accordance with the guidelines of the American and European Societies of Echocardiography for cardiac chamber quantification^(15)^. The standard echocardiographic views were obtained with a 3.5-MHz transducer for all subjects.

### Statistical Analysis

The averages values of SFCT, pRNFLT, MD, PSD, OPP, IOP, and AL for both the eyes of each subject were processed. All statistical analyses were performed using the Statistical Package for the Social Sciences (SPSS version 22; Chicago, IL, USA). The normality of the data was confirmed using the Kolmogorov-Smirnov test (p<0.05). Continuous variables were expressed as mean value ± standard deviation (SD). An independent student’s t-test and analysis of variance (ANOVA) were applied to compare the variables among the groups. For the sample size calculation, 38 patients with 30-50% LVEF, 34 with CHF but <30% LVEF, and 40 healthy individuals from each group were included in the determination of 2-point difference in determining appropriate number (DAN) scale, with a power of 80% and a significance level of 1%. The categorical variables among the groups were analyzed by using the chi-square test. Pearson’s correlation was applied to examine the relationships among the measured variables. All results were considered to be statistically significant at p=0.05.

## RESULTS

A total of 38 eyes of 38 patients with CHF and 30-55% LVEF (group 1) and 34 eyes of 34 patients with CHF and <30% LVEF (group 2) were included in this study, and their data were compared with those of 40 ageand sex-matched healthy eyes of 40 control subjects. The mean age of group 1 patients was 64.63 ± 5.43 years, that of group 2 patients was 63.00 ± 3.16 years, and that of control group subjects was 64.08 ± 2.84 years. No significant differences were noted in the values of refractive error, IOP, OPP, MD, PSD, AL, age, and sex among the 3 groups. The demographic and clinical information of the patients and control subjects are given in table 1.

**Table 1:** Demographic and clinical data of the study population

	Group 1 (n=38)	Group 2 (n=34)	Control (n=40)	p-value
Mean Age (year)	64.6 ± 5.4	63.0 ± 3.1	64.1 ± 2.8	0.46^*^
Sex Female	22 (57.8%)	24 (70.5%)	25 (62.5%)	0.58^**^
Male	18 (42.2%)	16 (29.5%)	15 (37.5%)	
IOP (mmHg)	15.3 ± 1.5	16.8 ± 1.8	17.9 ± 1.7	0.67^*^
AL (mm)	22.9 ± 0.71	23.06 ± 0.6	23.4 ± 0.8	0.72^*^
PSD (dB)	1.61 ± 0.07	1.66 ± 0.11	1.55 ± 0.09	0.41^*^
MD (dB)	-0.81 ± 0.05	-0.86 ± 0.09	-0.76 ± 0.07	0.061^*^
SBP	114.2 ± 12.6	110.7 ± 11.3	118.6 ± 11.8	0.22^*^
DBP	83.5 ± 9.7	82.3 ± 10.3	87.2 ± 10.8	0.17^*^
OPP	47.1 ± 8.5	45.02 ± 6.7	47.27 ± 8.2	0.07^*^

Data are presented as mean ± standard deviation. IOP= Intraocular pressure; AL= Axial length; DBP= Diastolic blood pressure; MD= Median deviation; PSD= Pattern standard deviation; OPP= Ocular perfusion pressure; SBP= Systolic blood pressure

* One-way ANOVA;

** Chi-square test.

**Figure 1:**
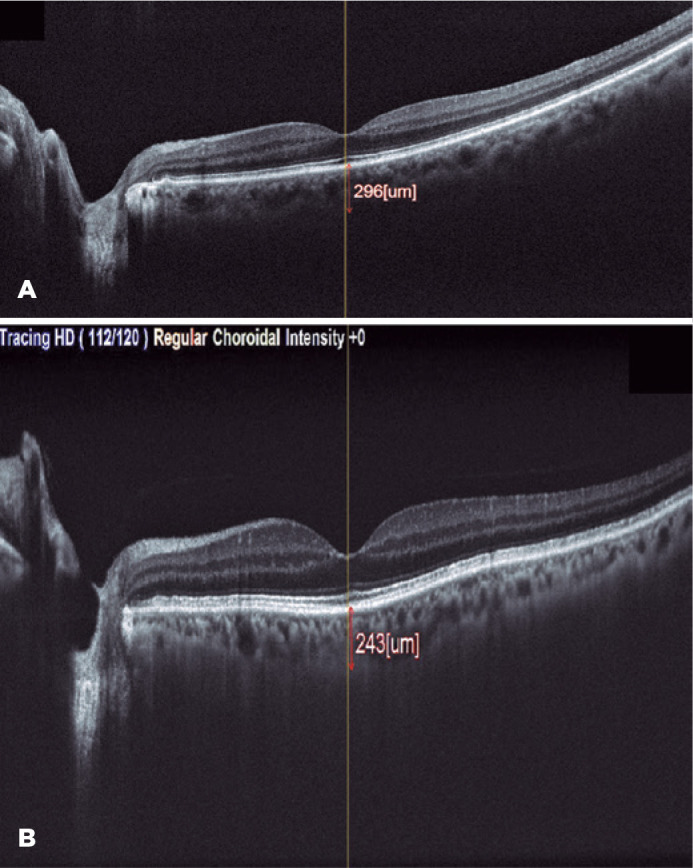
A) SFCT measurement with spectral domain optic-coherence tomography in a healthy control. B) SFCT measurement in a patient with 30-50% LVEF value (Group 1).

**Figure 2:**
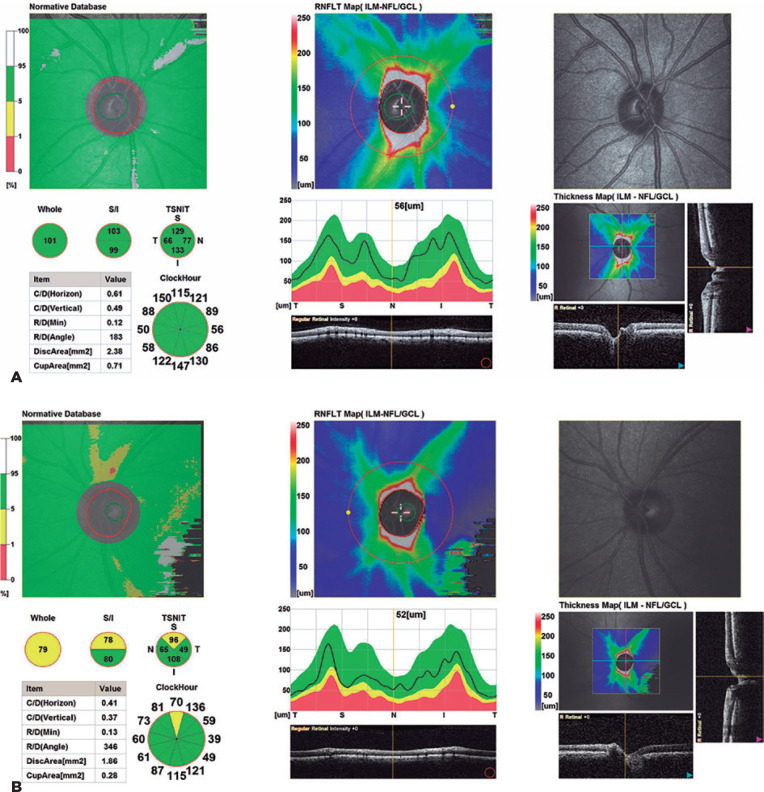
A) The pRNFLT measurement in a healthy control, B) RNFL thickness in a patient with <30% LVEF value (Group 2).

In the SFCT evaluation, statistically significant difference was noted among the groups 1, 2, and healthy controls (p<0.001). In post-hoc analysis, the mean SFCT was 250.24 ± 68.34 µm in group 1 and 216.72 ± 71.24 µm in group 2; this difference was statistically significant. The mean SFCT in the control group was 273.64 ± 77.68 µm, which was significantly greater than that in groups 1 and group 2. Figures 1A and 1B illustrate the SCFT images from a healthy control and a group 1 patient. The average pRNFLT was 100.34 ± 8.24 µm in group 1, 95.44 ± 6.67 µm in group 2, and 102.34 ± 8.24 µm in the control group. Figures 2A and 2B depict the pRNFLT images of 1 subject each from the control group and from group 2. The mean pRNFLT was significantly lesser in group 2 patients than in group 1 patients and the control subjects (p=0.042 and p=0.036, respectively), albeit the difference in the thicknesses between group 1 and the control group was not statistically significant. Analyses of the pRNFL quadrants parameters revealed that all quadrant thicknesses in group 2 were significantly lesser than those in group 1 patients and control subjects. The comparative analyses are depicted in table 2.

**Table 2:** Summary of statistical analyses for comparison of SCFT, pRNFLT (average), and quadrants of pRNFLT

	Group 1	Group 2	Control	p-value
SFCT µm	250.24 ± 68.34^a,b^	216.72 ± 71.24^c^	273.64 ± 77.68	0.001^*^
p RNFLT (average) µm	100.34 ± 8.24^a^	95.44 ± 6.67^c^	102.34 ± 8.24	0.02^*^
p RNFLT-superior µm	113.22 ± 8.45^a^	106.47 ± 5.47^c^	115.78 ± 4.23	0.01^*^
p RNFLT-inferior µm	111.50 ± 8.76^a^	106.08 ± 5.10^c^	115.00 ± 6.68	0.01^*^
p RNFLT-nasal µm	88.19 ± 5.73^a^	83.81 ± 5.88^c^	90.07 ± 7.93	0.02^*^
pRNFLT-temporal µm	88.06 ± 5.66^a^	83.72 ± 6.37^c^	89.43 ± 6.67	0.03^*^

Data are presented as mean ± standard deviation. SFCT= Subfoveal choroidal thickness; p RNFL= Peripapillary retinal nerve fiber layer;

* = One-way ANOVA test Post-hoc analysis (Tukey).

a = statistical difference between Groups 1 and 2.

b = statistical difference between Group 1 and Control.

c = statistical difference between Group 2 and Control.

The correlation analysis was performed for the factors age, AL, IOP, LVEF, OPP for SFCT, and pRNFLT and the quadrants of pRNFLT values in the CHF group. Statistically significant correlation was noted between the SFCT and the values of LVEF and age (r=0.498, p=0.03, and r=-0.346, p=0.044, respectively). In addition, significant correlation was noted between pRNFLT and age (r=-0.341, p=0.043). However, no significant correlation was recorded between the pRNFLT and LVEF (r=0.31, p=0.46).

Significant univariate correlations in the presence of CHF are listed under table 3. The receiver operating characteristics (ROC) analysis revealed that a SFCT of ≤250 µm can predict the presence of CHF with a sensitivity of 85%, specificity of 69.1%, positive predictive value of 26%, and negative predictive value of 81% (AUC = 0.80, p<0.001). The ROC analysis also revealed that pRNFLT of ≤97 µm (sensitivity: 97.5%, specificity: 49.5%, positive predictive value: 34.0%, and negative predictive value: 93%) indicates the presence of CHF. Multivariate logistic regression analysis was accordingly performed to demonstrate the independent predictors of CHF in our study groups (Table 3).

**Table 3:** Univariate and multivariate analyses for selected clinical and SD-OCT variables toward determining CHF

	Univariate logistic regression			Multivariate logistic regression	
	p-value	OD	95% CI	p-value	OR 95% CI
SFCT <250 µm	<0.001	12.07	4.44-32.80	<0.001	8.77 3.00-25.64
pRNFLT <97 µm	<0.001	36.89	4.80-283.16	0.002	25.5 3.17-206.02
Gender (Male)	0.65	1.19	0.55-2.60		
Age	0.59	1.02	0.93-1.12		

SFCT= Subfoveal choroidal thickness; p RNFL= Peripapillary retinal nerve fiber layer; OD= odds ratio; CI = confidence interval.

## DISCUSSION

We evaluated the SFCT and pRNFLT in patients with CHF and compared these values with those of healthy controls. The major findings of the present study are as follows: (1) the mean SFCT was significantly lower in both the CHF groups in comparison with that in the control group. Post-hoc analysis confirmed that the mean SFCT was significantly lower in patients with <30% LVEF than in those with 30-50% LVEF. (2) The average pRNFLT was significantly lower for patients with <30% LVEF as compared with the other 2 groups.

Several recent studies have evaluated the choroidal thickness in different ocular or systemic diseases^(16-18)^. Because the choroidal blood flow is responsible for nourishing the outer segment of the retina, it plays an important role in the photoreceptor metabolic processes. A few studies have evaluated the effect of cardiovascular diseases on the choroidal thickness. For example, Ahmad et al. reported that the SFCT was thinner in patients with a history of coronary artery diseases than in healthy controls, which indicates that these findings may predispose these patients to age-related macular degeneration^(12)^. A recent study revealed that the values of superficial and deep fovea, vessel density in all retinal/choroidal layers, and choroidal flow area decreased in optical coherence tomography angiography before any clinical fundus sign were noted in patients with coronary artery diseases, and the authors added that retinal and choroidal microvasculature changes were closely related to the presence of coronary artery and branch stenosis^(19)^.

Similarly, Altinkaynak et al. revealed that the SFCT was lower in the eyes of CHF patients compared to that in the eyes of healthy controls and also that the thinning of the SFCT was correlated with a decrease in the LVEF levels. Although these authors did not specifically evalua te the RNFL, they suggested that their findings may be associated with a higher risk of development of various chorioretinal diseases, such as diabetic retinopathy^(20)^. Previous studies have shown that the choroidal thickness decreases in patients with diabetes mellitus and that the underlying choroidal vasculature changes may be associated with the onset or progression of diabetic retinopathy^(21)^. The choroidal thinning associated with diabetes mellitus may also be related to tissue ischemia and hypoxia. Moreover, it is known that the coexistence of diabetes mellitus and heart failure is quite high. Therefore, the knowledge about the presence of additional CHF may be important for the management of diabetic retinopathy considering that CHF may affect the progression of this retinopathy.

To the best of our knowledge, this is the first study to evaluate the pRNFLT in patients with CHF. The RNFL thickness is a quantitative evaluation of the viable ganglion cells in the axonal mass, and any alterations in the RNFL can now be easily detected with the development of OCT. In clinical practice, the RNFL and SAP measurements are generally applied for the diagnosis and follow-up of glaucoma. Several studies have hypothesized that optic nerve perfusion may be responsible for the neurodegeneration observed in glaucoma and also that a reduction in the optic nerve head perfusion may be associated with glaucoma^(22,23)^. A previous study demonstrated reduced diastolic velocity and increased resistance index in the ophthalmic artery of patients with CHF and also indicated that CHF could be a risk factor for low ocular perfusion, which is considered to be a risk factor for glaucoma^(8)^. In our study, we noted no statistical differences among the 3 groups with respect to their mean TD and PD values of SAP. The loss in RNFL thickness was detected in only group 2, whereas group 1 and the control group showed similar RNFL thicknesses. The group 2 patients, who had a lower LVEF, may also have a lower optic nerve perfusion pressure. The loss of RNFL thickness in group 2 may therefore be associated with their lower LVEF value. It is well known that, progressive RNFL thinning is predictive of functional decline in glaucoma patients and that the pRNFLT has a greater sensitivity than SAP test in the eyes with early glaucoma, but not in those with moderate to advanced level of the disease^(24)^. We believe that our findings may be important in pre-perimetric glaucoma detections for patients with CHF. Freitas et al. evaluated the association between CHF and optic nerve head alterations and found that CHF is associated with lower OPP and glaucomatous optic nerve head changes. However, they did not evaluate RNFL with OCT, rather they used confocal scanning laser ophthalmoscopy and found that the Moorfields regression analysis was outside the normal limits in 27.6% eyes of the CHF patients, with 10% frequency of glaucoma in the CHF group^(25)^. However, Lamparter et al. evaluated the association between ocular, cardiovascular, morphometric, and lifestyle parameters and the RNFL thickness and found no relationship between the RNFL and cardiovascular diseases. Nevertheless, they did not indicate whether the subjects had CHF^(26)^.

In correlation analyses, we found that the SFCT values were statistically significantly associated with the LVEF, while the pRNFLT values were not (r=0.498, p=0.03 versus r=0.31, p=0.46). Our results are well-correlated with those of a previous study. Altinkaynak et al. also demonstrated that the thinning of the SFCT was correlated with a decrease in the LVEF levels^(20)^. Our findings may thus be explained by the vasoconstriction and chronic ischemia mechanisms. For instance, vasoconstriction may develop in the orbital and choroidal vessels in response to low cardiac output, and, consequently, the SFCT and pRNFLT may be lower. Chronic tissue ischemia caused by vasoconstriction in patients with CHF and RPE atrophy and retinal nerve loss occur as a secondary concern, which explains the reason for the low SFCT and pRNFLT. In addition, we found that SFCT <250 µm and pRNFLT <97 µm acted as independent predictors of CHF in multivariate analyses. These SD-OCT parameters are frequently applied in the diagnosis follow-up and progression analyses of several diseases. Therefore, it should be remembered that, chorioretinal diseases, glaucoma, and optic nerve diseases may develop or progress in patients with CHF.

Our study has some limitations. For instance, the size of the study groups were relatively small. In addition, a manual method was used to measure SFCT instead of using an automated software program, which is more reliable. Moreover, only SD-OCT was performed, with the evaluation of the optic disc perfusion. We believe that the use of ultrasound and/or optical coherence tomography angiography would be more appropriate for studying perfusion in the future studies.

In conclusion, we found that the SFCT and the pRNFLT decreased in accordance with the extent of heart failure. In clinical practice, the knowledge about a patient’s heart failure status when approaching a chosen treatment for the chorioretinal disease is believed to increase the effectiveness of the follow-up and the prognosis of the disease.

## References

[1] Remme WJ, Swedberg K, Task Force for the Diagnosis and Treatment of Chronic Heart Failure,
European Society of Cardiology (2001). Treatment of Chronic Heart Failure ESoC. Guidelines for the
diagnosis and treatment of chronic heart failure. Eur Heart J.

[2] Savarese G, Lund LH. (2017). Global public health burden of heart failure. Card Fail Rev.

[3] Marti CN, Gheorghiade M, Kalogeropoulos AP, Georgiopoulou VV, Quyyumi AA, Butler J. (2012). Endothelial dysfunction, arterial stiffness, and heart
failure. J Am Coll Cardiol.

[4] Petrie JR, Guzik TJ, Touyz RM. (2018). Diabetes, hypertension, and cardiovascular disease: clinical
insights and vascular mechanisms. Can J Cardiol.

[5] Almeida OP, Flicker L. (2001). The mind of a failing heart: a systematic review of the
association between congestive heart failure and cognitive
functioning. Intern Med J.

[6] Roy B, Woo MA, Wang DJ, Fonarow GC, Harper RM, Kumar R. (2017). Reduced regional cerebral blood flow in patients with heart
failure. Eur J Heart Fail.

[7] Choi BR, Kim JS, Yang YJ, Park KM, Lee CW, Kim YH (2006). Factors associated with decreased cerebral blood flow in
congestive heart failure secondary to idiopathic dilated
cardiomyopathy. Am J Cardiol.

[8] Almeida-Freitas DB, Meira-Freitas D, Melo LA Jr, Paranhos A Jr, Iared W, Ajzen S (2011). Color Doppler imaging of the ophthalmic artery in patients with
chronic heart failure. Arq Bras Oftalmol.

[9] Cao J, McLeod S, Merges CA, Lutty GA. (1998). Choriocapillaris degeneration and related pathologic changes in
human diabetic eyes. Arch Ophthalmol.

[10] Cavallotti C, Corrado BG, Feher J. (2005). The human choriocapillaris: evidence for an intrinsic regulation
of the endothelium?. J Anat.

[11] Shi R, Zhao L, Qi Y. (2018). The effect of fenofibrate on early retinal nerve fiber layer loss
in type 2 diabetic patients: a case-control study. BMC Ophthalmol.

[12] Ahmad M, Kaszubski PA, Cobbs L, Reynolds H, Smith RT. (2017). Choroidal thickness in patients with coronary artery
disease. PLoS One.

[13] Balmforth C, van Bragt JJ, Ruijs T, Cameron JR, Kimmitt R, Moorhouse R (2016). Chorioretinal thinning in chronic kidney diseas e links to
inflammation and endothelial dysfunction. JCI Insight.

[14] Gherghel D, Orgül S, Gugleta K, Gekkieva M, Flammer J. (2000). Relationship between ocular perfusion pressure and retrobulbar
blood flow in patients with glaucoma with progressive damage. Am J Ophthalmol.

[15] Lang RM, Badano LP, Mor-Avi V, Afilalo J, Armstrong A, Ernande L (2015). Recommendations for cardiac chamber quantification by
echocardiography in adults: an update from the American Society of
Echocardiography and the European Association of Cardiovascular
Imaging. J Am Soc Echocardiogr.

[16] Kılıç R, Kurt A, Acer E, Öktem Ç, Kocamış Ö. (2017). Choroidal thickness in psoriasis. Int Ophthalmol.

[17] Steiner M, Esteban-Ortega MDM, Muñoz-Fernández S. (2019). Choroidal and retinal thickness in systemic autoimmune and
inflammatory diseases: A review. Surv Ophthalmol.

[18] Yiu G, Chiu SJ, Petrou PA, Stinnett S, Sarin N, Farsiu S (2015). Relationship of central choroidal thickness with age-related
macular degeneration status. Am J Ophthalmol.

[19] Wang J, Jiang J, Zhang Y, Qian YW, Zhang JF, Wang ZL. (2019). Retinal and choroidal vascular changes in coronary heart disease:
an optical coherence tomography angiography study. Biomed Opt Express.

[20] Altinkaynak H, Kara N, Sayın N, Güneş H, Avşar S, Yazıcı AT. (2014). Subfoveal choroidal thickness in patients with chronic heart
failure analyzed by spectral-domain optical coherence
tomography. Curr Eye Res.

[21] Wang XN, Li ST, Li W, Hua YJ, Wu Q. (2018). The thickness and volume of the choroid, outer retinal layers and
retinal pigment epithelium layer changes in patients with diabetic
retinopathy. Int J Ophthalmol.

[22] Flammer J, Orgül S, Costa VP, Orzalesi N, Krieglstein GK, Serra LM (2002). The impact of ocular blood flow in glaucoma. Prog Retin Eye Res.

[23] Bojikian KD, Chen CL, Wen JC, Zhang Q, Xin C, Gupta D (2016). Optic disc perfusion in primary open angle and normal tension
glaucoma eyes using optical coherence tomography-based
microangiography. PLoS One.

[24] Triolo G, Rabiolo A. (2020). Optical coherence tomography and optical coherence tomography
angiography in glaucoma: diagnosis, progression, and correlation with
functional tests. Ther Adv Ophthalmol.

[25] Meira-Freitas D, Melo LA Jr, Almeida-Freitas DB, Paranhos A Jr (2012). Glaucomatous optic nerve head alterations in patients with
chronic heart failure. Clin Ophthalmol.

[26] Lamparter J, Schmidtmann I, Schuster AK, Siouli A, Wasielica-Poslednik J, Mirshahi A (2018). Association of ocular, cardiovascular, morphometric and lifestyle
parameters with retinal nerve fibre layer thickness. PLoS One.

